# Novel insights into rural spatial design: A bio-behavioral study employing eye-tracking and electrocardiography measures

**DOI:** 10.1371/journal.pone.0322301

**Published:** 2025-05-02

**Authors:** Minqi Shi, Ruili Wang, Lei Zhang

**Affiliations:** Jingjinji Spatial Intelligent Perception Collaborative Innovation Center, Hebei University of Engineering, Handan, China; Anhui Polytechnic University, CHINA

## Abstract

In order to objectively assess the effectiveness of rural space design on the affective dimension, this study utilized eye-tracking and electrocardiogram (ECG) monitoring techniques to quantify users’ visual attention and emotional responses and to assess the impact of rural design on users’ affective experience. The results show that incorporating natural elements and cultural features into the design enhances the values of the subjects’ pupil diameter change rate, Heart Rate Variability Index (HRV), subjective evaluation and reduces their Saccade Velocity Average. The experimental results not only verify the application value of eye tracking and ECG monitoring techniques in assessing the design effect of rural space, but also provide a scientific assessment method based on the user’s physiological and emotional responses, thus providing a strong support for the optimization of rural space design.

## Introduction

The importance and urgency of rural spatial planning and the enhancement of residential environments have become increasingly evident, as they are critical to ecological balance and sustainable social development. These aspects serve as core elements in implementing the rural revitalization strategy and promoting urban-rural integration. In research on improving rural living environments, establishing a human-centered value system that encompasses key processes such as cognition, judgment, evaluation, planning, design, prediction, and feedback is essential for rural development [1]. Among these processes, uncovering users’ objective perceptions and comprehensively considering the universal needs of different user groups are crucial for rural design. Environmental evaluation revolves around the aesthetic experience of individuals, with some scholars arguing that aesthetic perception is a form of emotional response [2]. Throughout human evolution, a specific mode of environmental cognition, driven by physiological needs, has gradually developed. In rural environments, physiological needs exert a more direct influence on human cognition. Studies have shown that individuals’ preferences for natural environments may be closely linked to deeper survival instincts [3]. This cognition is often reflected in subjective judgments, objective physiological responses, and behavioral changes [4]. Existing evaluation studies predominantly employ subjective assessments and post-occupancy evaluations to analyze environmental cognition. Using method of semantic differential, researchers collect linguistic data, design surveys, conduct rating experiments, and perform factor analyses to convert subjective evaluations into quantitative data. This approach enables the quantitative analysis of affective responses to architecture and its spatial environments [5].

However, changes in perception are complex and multidimensional, often difficult to fully describe using language alone. Although the quantification of subjective evaluations has improved accuracy and reliability, the limitations of these methods become particularly apparent when communication barriers arise due to differences in educational background or language. Moreover, architects and planners, lacking the cultural and linguistic immersion of living in rural areas, often find it challenging to grasp the needs of villagers and identify critical points for intervention in rural design. To address these challenges, more accurate and appropriate methods are needed. With the advancement of quantitative data research and innovations in cognitive science and bio-signal acquisition technologies, new tools have emerged to quantify perception. Emotional Engineering, a user-centered research approach, utilizes technologies such as eye-tracking, facial recognition, and Electrocardiograms (ECG) to understand and capture users’ emotional needs, translating these needs into specific design elements or technical parameters to enhance user satisfaction and emotional value [6]. Emotional Architecture, as an interdisciplinary field, integrates the core principles of Emotional Engineering and architecture. By leveraging advanced cognitive science and bio-signal acquisition technologies, it delves into human perception and emotional responses to architectural spaces, aiming to optimize environmental design [7]. For instance, eye-tracking technology automatically detects and records visual behavior data, uncovering how individuals perceive and interact with their surroundings [8]. This technology effectively analyzes how people search, select, and engage with an environment, providing valuable insights for spatial planning, emergency evacuation, and user experience enhancement. Recent studies using eye-tracking experiments have shown that green infrastructure can significantly promote cognitive recovery [9]. Additionally, training with eye-tracking modeling has been demonstrated to improve construction workers’ hazard recognition performance. While eye-tracking technology has accumulated empirical results in fields such as architectural environment perception and construction safety [10], its application in rural contexts remains limited. Tingting Su et al., for example, used eye-tracking technology combined with subjective evaluations and objective data to quantitatively analyze user preferences for public spaces in rural Beijing, offering empirical support for theories of rural landscape preference [11]. In addition to eye-tracking, ECG technology serves as another key bio-signal acquisition tool, widely used in medicine to record and analyze heart activity. Recently, researchers have begun employing traditional ECG techniques to evaluate the emotional stress caused by thermal environments in buildings [12]. Environmental factors such as green view index have also emerged as significant topics of study regarding their effects on emotional stress [13]. However, traditional contact-based ECG methods, which rely on skin electrodes to capture heart activity, may cause discomfort to participants, potentially affecting data accuracy. With technological advancements, non-contact ECG detection has emerged, using sensors that monitor cardiac activity without direct skin contact, thus providing a more comfortable method for physiological state monitoring [14]. Studies have shown that non-contact ECG technology is widely applied in cardiac diagnostics and health monitoring [15], and it offers robust quantitative data for assessing the impact of architectural environments on health and emotional well-being [16]. This technology provides new methodologies and applications for evaluating emotional feedback and psychological comfort in architectural spaces. In particular, it enables the precise capture of users’ emotional changes, offering scientific evidence to inform architectural design. In summary, while eye-tracking and ECG technologies have gained attention in the evaluation of architectural environments, research focusing on rural contexts remains scarce.

To further analyze the application of emotion quantification technologies in rural environments, prior studies have conducted experimental analyses of real rural settings based on eye-tracking technology. These studies explored differences in eye-tracking data between villagers and tourists in real-world environments. The findings revealed that visual attractiveness is influenced not only by greenery coverage but also by variations in spatial enclosure [17]. Building upon previous research, this study aims to establish a method for evaluating rural design schemes by analyzing eye-tracking data and non-contact ECG data to assess users’ emotional responses to environmental design. The specific studies are as follows:

i. How can eye-tracking data and contactless electrocardiographic data be integrated to establish an effective evaluation method for emotional responses to rural environmental design?
ii. Based on the patterns of emotional responses and physiological indicators, how can the analysis results be translated into actionable rural environmental design strategies to enhance users’ sense of safety, fulfillment, and well-being?

## Materials and methods

### Subjects

In order to ensure the scientific validity of the experimental process and the accuracy of the data, G power 3.1 software was used in this study to calculate the required sample size. According to Cohen’s research, an experimental design is considered to have high detection power when the statistical power (1-β) exceeds 0.8 [18]. To ensure sufficient statistical power, the Effect Size(d_z_) is typically set to 0.5, with a significance level (α) of 0.05. Calculations indicate that a minimum of 14 participants is required to achieve a statistical power of at least 0.8.

Rigorous screening criteria were adopted in this study to ensure the consistency and accuracy of subjects during eye tracking and ECG data collection, aiming to exclude individual differences and external factors that may affect the quality of data and the reliability of experimental results. The screening criteria for the subjects included: the ability to think positively, clear awareness of external stimuli, and clear expression; and naked or corrected visual acuity of 1.0 or higher without visual defects such as color blindness, color weakness, or ocular diseases. In addition, this study strictly adhered to the ethical guidelines to ensure that the experiment did not pose any hazardous risks to the subjects, and written informed consent was obtained from all subjects. Subjects had the right to withdraw from the experiment at any time during the experiment, and the experimental protocol was ethically reviewed by the Medical Ethics Committee of the Affiliated Hospital of Hebei University of Engineering, with the approval number 2023[K]110. After recruitment and screening 30 undergraduate as well as masters students (10 males and 20 females) aged 20–30 years were finally selected as subjects for this experiment and the information is shown in Table 1.

**Table 1:** Subjects’ information.

Gender	Number of persons	Percentage	Education level	Number of persons	Percentage	Age
Male	10	33.33%	Undergraduate	6	20%	20-30
Female	20	66.66%	Postgraduate	24	80%	

### Stimulus and element design

The study and delineation of rural space types can be discussed from various perspectives, such as function, morphology and economic activities. In the design of rural space, street space, residential courtyard and node space play an important role [19]. Street space is not only the transportation network connecting all parts of the village, but also an important place for villagers’ daily life and social interaction. The residential courtyard is the basic unit of village space, reflecting local characteristics and cultural traditions. Nodal spaces, such as squares and bazaars, are the centers of villagers’ public activities and have an important influence on the social structure and cultural life of the village. Therefore, the study of street space, residential courtyard and nodal space in villages can help to comprehensively understand and promote rural revitalization, protect the cultural characteristics of villages and improve the quality of life of rural residents.

In order to better design the rural environment to meet the subjects’ emotional needs for the rural environment. The rural design strategy should comprehensively consider regional characteristics, ecological sustainability, cultural heritage and modern aesthetics, and create a rural landscape that is both distinctive and harmonious through a unified interface style, reasonable vegetation configuration and implantation of cultural symbols [20]. In this empirical study, the following three aspects are used to enhance the rural space and environment.

Interface landscape unity: a unified design language is used to guide the architectural style and ensure the coordination and harmony between different buildings, thus enhancing the overall visual aesthetics. At the same time, careful adjustment of interface elements such as colors, materials and decorations can not only enhance the attractiveness of the countryside, but also stimulate pedestrians’ interest in exploring and enhancing their perception and appreciation of the rural environment.

Rational Configuration of Vegetation: Rational Layout of Green Plants: Green plants are introduced in streets and public spaces to create green space and provide visual comfort and ecological value. When carrying out the layout of greenery, emphasis is placed on realizing the guidance of flow, aiming to create spaces that are both visually appealing and able to meet functional needs through the rational configuration of plants.

Integration of Cultural Symbols: Emphasizing the principle of regionalism, rural design should delve deeply into and preserve local historical and cultural heritage. These cultural elements can be innovatively incorporated into landscape design, enhancing the cultural identity and appeal of rural areas. This approach ensures that traditional culture is both preserved and revitalized within modern design.

**Fig 1:**
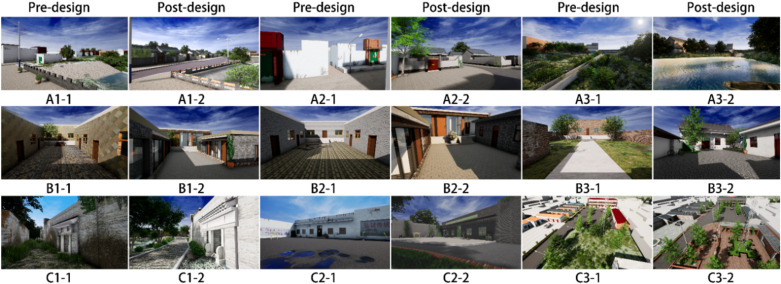
Stimuli used in the experiment.

It has been shown that photographs have a positive effect on brain and autonomic nervous system activities [21], so the village spatial scenes were transformed into virtual scenes by modeling replica and remodeling design. A total of nine spatial scenes (A street space, B residential compound, C node space) in Nanlizhuang village were selected for the experiment. It was ensured that the height of each viewpoint and the viewpoints before and after the design remained consistent to maintain the coherence of the design and enhance the user experience. The experimental stimuli were presented using photographs with a resolution of 2560 × 1440 (Fig 1).

### Eye tracking and non-contact ECG measurement devices and methods

#### Eye tracking.

Visual behavior is one of the behaviors that can best reflect human psychological activities, and with the help of quantitative analysis of visual behavior one can interpret human attention allocation and preference. The eye-tracking data of this experiment were measured using the aSee Pro desktop eye-tracking system developed by China’s 7invensun, a telemetric gaze point tracking algorithm based on a three-dimensional model of the human eye. The device uses low-power infrared light to illuminate the human eye, enhancing the visibility of the pupil and corneal reflexes, which allows the system to more accurately locate the center of the cornea and the center of the pupil, and to extract eye movement characteristics. As a result, the aSee Pro Desktop Eye Tracker allows continuous tracking of the subject’s eye-tracking data while viewing a picture of the stimulus played on a 21” screen at a distance of 60 cm. The color monitor has a screen resolution of 1920x1080, and the desktop eye tracker has a sampling frequency of 140 Hz. During the eye tracking experiment, the subject does not have to wear any equipment, and the eye’s position and trajectory on the screen can be accurately captured without the need for any constraints. This ensures a non-invasive and natural state of the subject during the experiment and improves the accuracy of the data.

#### Non-contact ECG.

Fluctuations in ECG data can intuitively reflect the activity status of the sympathetic and parasympathetic nerves in the human body, which in turn provides an effective biological indicator for analyzing an individual’s emotional state. In psychophysiology studies, the balance between sympathetic and parasympathetic nerves is crucial for maintaining emotional stability. Capturing a subject’s facial expression through a camera provides access to rich nonverbal information that is closely related to a person’s emotional state. Non-contact ECG technology is based on the ability of sophisticated remote photoplethysmography (rPPG) to analyze changes in light intensity caused by blood flow and indirectly obtain ECG parameters such as heart rate and heart rate variability. Therefore, this empirical study used a 4-megapixel high-speed camera to record the subjects’ facial expressions, quantify the light intensity reflected from the subjects’ faces, and measure the subjects’ ECG signals with the help of the Heart ERS software developed by a team from Sangmyung University in South Korea through image processing techniques. In addition, the non-contact ECG technology does not require skin contact, which reduces interference with the subject, improves the comfort and convenience of data collection, and facilitates the assessment of an individual’s physiological responses and emotional state.

### Experimental procedures

**Fig 2:**
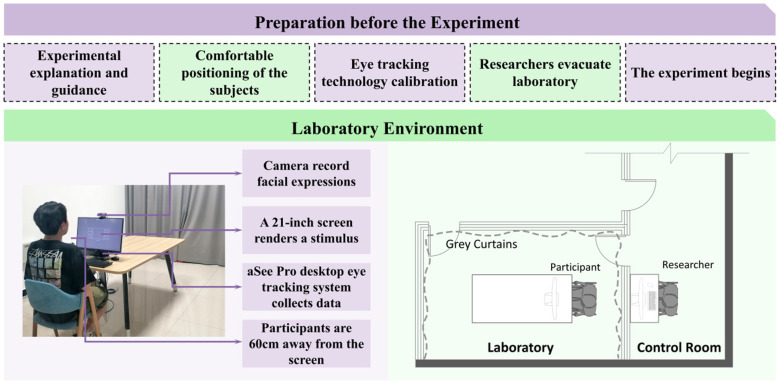
Pre-laboratory preparation and laboratory environment.

This experiment was successfully conducted from 21/06/2024–28/06/2024 in the Cognitive Data Laboratory of the International Center for Architecture and Emotion Research at Hebei University of Engineering. The experimental environment was strictly controlled in order to ensure the standardization of the experiment and reduce the interference of external variables. Each subject was subjected to the same closed and quiet laboratory conditions for about 30 minutes (Fig 2). To minimize the influence of individual differences on the results during the experiment, it was ensured that each subject viewed the same visual stimuli under consistent environmental conditions. The lighting, temperature and humidity in the laboratory were kept constant to exclude the potential influence of environmental factors on the experimental results.

Prior to the start of the experiment, the researchers explained the experimental procedures and precautions in detail to the subjects to ensure that each subject had a full understanding of and was prepared for the experimental requirements. To ensure the accuracy of the eye-tracking data and the reliability and validity of the experimental results. A 9-point calibration of each subject’s eye-tracking system was performed before presenting the stimuli, and the next step of the formal experiment could be performed only when the calibration accuracy reached more than 90%.

**Fig 3:**
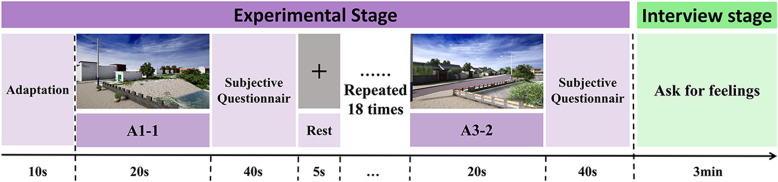
Flowchart of the experiment.

The experimental process is as follows (Fig 3).

1) Step 1: Scene Adaptation

Human perception of the environment is diverse, multifaceted, and multilayered. In order to guide subjects to enter the experiment in a positive state, this study includes a scene adaptation stage, where formal data collection begins after passing a 10s adaptation experience.

2) Step 2: Cognitive Experiment

Subjects were asked to view photos of the scene for 20s, during which they minimized physical activity and remained silent while the experimenter collected eye-tracking data and ECG data.

3) Step 3: Subjective Questionnaire

Subjects completed the subjective landscape comfort evaluation questionnaire based on the semantic differential analysis method within 40s after viewing, the experimental staff asked questions and scored records on the subjective questions, and the subjects made timely subjective evaluations.

4) Step 4: Inter-group Rest

After completing a group of scene viewing as well as questionnaire scoring, before the next group of scene experiments began, in order to avoid the fatigue and boredom feelings brought about by subjects being in the observation state for a long period of time, a 5s rest scene was set up between each group of experiments, which could eliminate the influence of the previous stimulus material on the measurement results.

### Data metrics extraction

This study comprehensively evaluated 30 sets of indicators, categorized into three main groups based on participants’ physiological and cognitive responses: eye-tracking data, ECG data, and subjective questionnaire data. The indicators and their scientific significance are as follows.

#### Eye-tracking data indicators.

Eye-tracking data indicators are eye-tracking data parameters extracted from eye tracking experiments. As shown in Table 2, indicators such as pupil diameter, fixation duration, average saccade velocity, and changes in areas of interest (AOI) reflect individuals’ visual attention patterns, physiological responses, and cognitive processes when processing visual information. The analysis of eye movement indexes facilitates the understanding of subjects’ visual attention patterns when viewing visual stimulus materials, thus providing a scientific basis for user experience research.

**Table 2:** Eye tracking metrics and their interpretation.

Eye-tracking data indicators	Interpretation of indicators
Average Pupil Diameter Change	Changes in pupil diameter are associated with cognitive load, emotional state, and difficulty in processing information. The mean pupil diameter change rate is a measure of the consistency with which the participants’ pupil size changes during the observation. Increases in this parameter may be associated with increased task difficulty or increased input of cognitive resources [22].
Fixation Duration	The Fixation Duration of each gaze that participants held while observing a visual stimulus. Fixation duration is an important parameter for measuring the depth and difficulty of visual processing, and longer fixation duration may indicate that participants performed deeper visual analysis or encountered cognitive challenges in a particular region [23].
Average Saccade Velocity	Saccade Velocity Average describes the fluctuation in the number of saccades made by participants within a certain timeframe. This metric reflects the efficiency of visual search strategies and the patterns of attention allocation when participants process visual information. A higher rate of variation may indicate rapid shifts in visual attention between different areas [24].
AOI	Specific areas of visual stimuli defined by the researcher to analyze the distribution and concentration of visual attention.The AOI setting allows the researcher to measure and assess eye-tracking metrics such as the participant’s point of fixation and fixation duration on specific visual content to infer the degree of attentional focus and the depth of visual processing [25].

#### Indicators of ECG data.

By analyzing Heart Rate Variability (HRV) in ECG data, the activity levels of the sympathetic and parasympathetic nervous systems can be assessed. For example, a lower HRV may indicate over-activation of the sympathetic nervous system, while a higher HRV may indicate an active state of the parasympathetic nervous system. In the fields of psychology and medicine, measures of HRV are used to assess stress levels, emotional states, cardiovascular health, and the effectiveness of therapeutic interventions. For example, meditation, relaxation training, and certain medications may increase HRV, whereas stress, anxiety, and certain diseases may decrease HRV [26]. HRV is the magnitude of change in the number of heart beats in response to human activity and is an indicator of cardiac autonomic function, with a normal reference range of 141 plus or minus 39 milliseconds, typically 102–180 milliseconds [27].

In this study, we used time domain measurements to measure HRV data during R-R intervals, and the specific metrics included two kinds of SDNN and RMSSD, which were calculated by the following formulas:


$$SDNN=\sqrt{\frac{1}{n}\sum_{i=1}^{N}{\left(t_{RR,i}-\overset{―}{t_{RR}}\right)}^{2}}$$

SDNN is calculated as the standard deviation of the R-R intervals, where N is the total number of normal beats, $t_{RR,i}$ is the R-R interval, and $\overset{―}{t_{RR}}$ is the average of the intervals of N beats. The greater the standard deviation, the higher the HRV, and vice versa. Normal values are greater than 100 milliseconds, but values less than 50 milliseconds are considered abnormal and indicate a significant decrease in HRV.


$$RMSSD=\sqrt{\frac{1}{N-1}\sum_{i=1}^{N-1}{\left(t_{RR,i+1}-t_{RR,i}\right)}^{2}}$$

RMSSD is calculated as the root-mean-square of the difference between two adjacent R-R intervals, where $t_{RR,i}$ and $t_{RR,i+1}$ are the lengths of two adjacent sinus cardiac cycles RMSSD is used to estimate the components of short-range HRV, with a normal value of (27 ± 12) ms.

#### Subjective questionnaire indicators.

During the data collection phase, special attention was paid to nine specific sets of scene modifications and participant feedback was recorded before and after these scene modifications. A subjective preference questionnaire based on pairs of positive and negative words was used to assess participants’ emotional responses to different scenes. The questionnaire design follows the hierarchical evaluation system proposed by Zhuang, which utilizes architectural concepts and vocabulary to semantically describe and modify the amount of information about the phase house of the spatial environment. Where each set of vocabulary pairs represents an evaluation dimension, positive vocabulary indicates a high-level evaluation of that dimension, while negative vocabulary indicates a low-level evaluation [28]. In order to achieve a more detailed emotional assessment, a five-level scoring system was set and 17 sets of adjective pairs were selected to construct the questionnaire [29]. As shown in Table 3, it is a subjective questionnaire for this empirical study, aiming to gain a deeper understanding of how scene modification affects people’s subjective feelings.

**Table 3:** Subjective questionnaire.

Imagine you’re in this scene and you feel						
Feeling calm	2	1	0	-1	-2	Feeling excited
Feeling comfortable	2	1	0	-1	-2	Feeling uncomfortable
Feeling special	2	1	0	-1	-2	Feelings of being everywhere
Feeling close	2	1	0	-1	-2	Feeling alienated
Feeling willing to stay	2	1	0	-1	-2	Wanting to pass quickly
Feeling spatially varied	2	1	0	-1	-2	Feeling monotonous in space
Feeling interesting	2	1	0	-1	-2	Boredom
Feeling natural	2	1	0	-1	-2	Feeling artificial
Feeling quiet	2	1	0	-1	-2	Feeling noisy
Feeling beautiful	2	1	0	-1	-2	Feeling ugly
Feeling friendly	2	1	0	-1	-2	Feeling unfriendly
Feeling bright	2	1	0	-1	-2	Feeling dim
Feeling stable	2	1	0	-1	-2	Feeling unstable
Feeling safe	2	1	0	-1	-2	Feeling unsafe
Feeling relaxed	2	1	0	-1	-2	Feeling aroused
Feeling positive	2	1	0	-1	-2	Feeling negative
Imagine you are in the scene and can you feel the emotions that come with the scene						
perceived discrimination	2	1	0	-1	-2	inability to feel

### Data analysis methods

**Fig 4:**
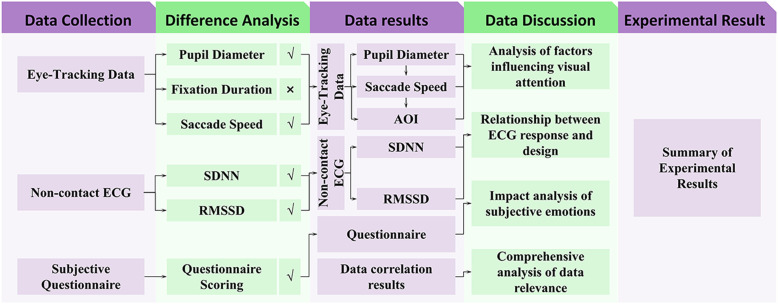
Data analysis methodology.

This study collected experimental data from 30 participants, including eye-tracking data, ECG monitoring data, and subjective questionnaire responses. The sampling rate for all participants exceeded 95%, meeting data quality standards and confirming the reliability and validity of the data. A systematic data analysis process was employed, involving precise data collection methods to obtain foundational experimental data. Additionally, differences and correlations were analyzed to evaluate significant variations under experimental conditions and the relationships among variables. The data analysis process encompassed the entire workflow, from data collection to statistical analysis (Fig 4).

To verify whether the data met the assumption of normal distribution and to select appropriate parametric statistical methods, this study conducted normality tests. Data processing was performed using Python 3.12.3 and PyCharm 2024.1.2, while statistical analyses were conducted using SPSS 26. The specific variables analyzed included pupil diameter of the left and right eyes before and after the intervention, fixation duration, average saccade velocity, SDNN, RMSSD, and subjective questionnaire metrics. Since the sample size was 30 (n = 30 < 50), the Shapiro-Wilk (S-W) test was used to assess data normality [30]. The results are shown in Table 4.

**Table 4:** Results of normality tests.

	Shapiro-Wilk Test		
Statistics	Degree of freedom	Significance
Difference pupil diameter of the left	0.930	30	0.050
Difference in pupil diameter of the right	0.956	30	0.240
Difference in fixation duration	0.825	30	0.000
Difference in saccade velocity average	0.573	30	0.000
SDNN Difference	0.963	30	0.358
RMSSD Difference	0.979	30	0.789
Difference in subjective questionnaire index	0.908	30	0.013

The results of the normality tests indicated that the p-values for the differences in left pupil diameter, right pupil diameter, SDNN, and RMSSD were all greater than or equal to 0.05 (p ≥ 0.05), suggesting that these variables followed a normal distribution. Therefore, paired-sample t-tests were deemed appropriate for the analysis of differences [31]. In contrast, the p-values for the differences in fixation duration, average saccade velocity, and subjective questionnaire metrics were all less than 0.05 (p < 0.05), indicating that these variables were non-normally distributed. As a result, the Wilcoxon signed-rank test was employed to assess the changes in these variables before and after the intervention [32].

## Results

### The effect of spatial design elements on visual attention

Spatial scenes (pre-design and post-design) were used as independent variables, while eye-tracking data, including left pupil diameter, right pupil diameter, fixation duration, and average saccade velocity, were treated as dependent variables. Paired-sample t-tests and Wilcoxon signed-rank tests were conducted for analysis. The results revealed significant differences in left pupil diameter (t = -3.188, p = 0.003), right pupil diameter (t = -3.479, p = 0.002), and average saccade velocity (Z = -2.705, p = 0.007). However, the change in fixation duration approached statistical significance (p = 0.098, p > 0.05), suggesting that no significant difference was observed for fixation duration. This may be attributed to the 20-second time limit for the presentation of each stimulus. Within such a short duration, participants were more likely to engage in quick scanning rather than prolonged fixation. Future studies may explore changes in fixation duration by extending the stimulus presentation time or modifying the experimental design.

To examine the specific changes in eye-tracking data before and after the design modification, box plots and line charts depicting the average pupil diameter change rate and average saccade velocity were generated using Origin 2022 software. The label “XX-1” represents the pre-design scene, while “XX-2” represents the post-design scene, with a total of 18 image materials compared across 9 sets of design modifications.

**Fig 5:**
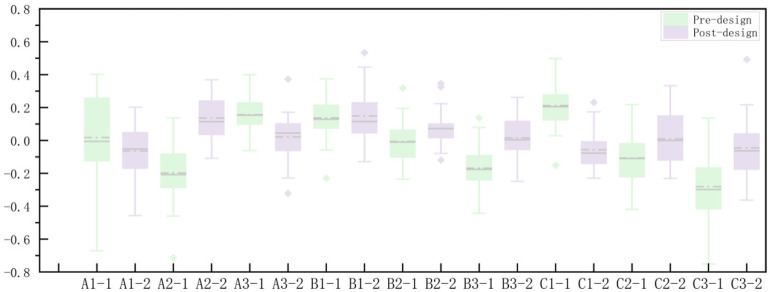
Box-and-Whisker plot and line chart of the average pupil diameter change rate.

The impact of pre- and post-design scene modifications on the participants’ average pupil diameter change rate was compared (Fig 5). The results revealed that the modification of six scenes significantly enhanced pupil responses. In 67% of the scene modifications, the post-design environments showed improvements in visual appeal, cognitive complexity, attention guidance, and emotional stimulation. This may be attributed to the post-modified environments, which, due to their aesthetic characteristics, evoked stronger emotional experiences and increased cognitive load. Participants were required to allocate more attention when processing visual information. Furthermore, the environmental modifications more effectively guided participants’ attention to focus on specific areas or objects, thereby influencing pupil responses.

**Fig 6:**
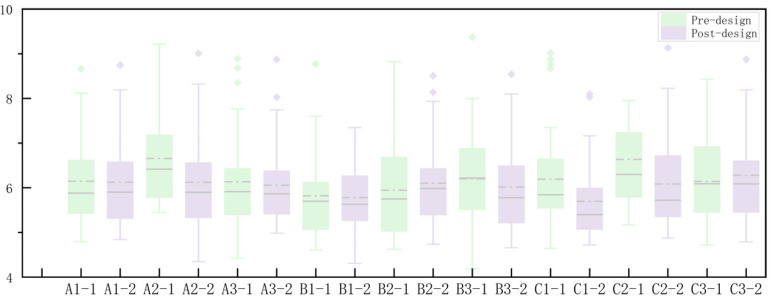
Box-and-whisker plot and line chart of the average saccade velocity.

The relationship between the participants’ average saccade velocity and the pre- and post-design scenes is shown (Fig. 6). In 89% of the scenes, the design modifications resulted in a decrease in the participants’ average saccade velocity, suggesting an improvement in attention. This may indicate that the participants experienced greater engagement and curiosity in the post-modified rural space, leading to stronger emotional reactions and deeper cognitive processing. Additionally, the integration of cultural symbols and adjustments to the spatial scale captured the participants’ attention, further enhancing their emotional experience. The design updates in the rural space positively contributed to the vitality of the rural environment.

**Fig 7:**
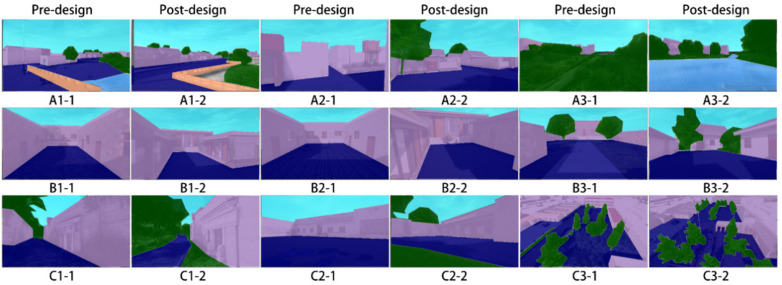
AOI division of eye-tracking data before and after scene design modifications.

To further investigate the participants’ attention to the design elements of the rural space, the aSeeStudio eye-tracking software was used to define AOI blocks for various design elements (e.g., ground, walls, water features, and landscape walls) in both pre- and post-design rural scenes [33]. Statistical analysis of the eye-tracking metrics within these AOI blocks revealed changes in the participants’ visual attention allocation. The division of participants’ visual focus areas when observing rural scenes at different design stages is presented (Fig 7), providing data to support the understanding of how design elements influence visual attention.

Based on the aforementioned AOI division, a systematic statistical analysis was further conducted on the eye-tracking metrics within each block. This quantifies participants’ visual attention to different design elements, revealing the specific impact of design modifications on the distribution of visual focus. By calculating the average pupil diameter, average saccade velocity, and average fixation duration for each AOI block, a deeper understanding of participants’ preferences and responses to the rural space design elements was obtained. The statistical results are presented in Table 5.

**Table 5:** Eye-tracking data changes before and after design modifications.

	Eye Movement Indicators	Floor	Walls	Sky	Water	Landscape Walls	Greenery	Units
**Pre-design**	Average Pupil Diameter	3.98	3.90	3.84	4.06	4.08	3.95	mm
	Saccade Velocity Average	1.34	2.11	0.39	0.29	0.59	0.97	px/ms
	Average Fixation Duration	0.56	0.64	0.27	0.11	0.38	0.51	s
**Post-design**	Average Pupil Diameter	3.96	3.96	3.91	3.97	3.90	3.97	mm
	Saccade Velocity Average	0.82	1.99	0.36	1.79	1.28	1.65	px/ms
	Average Fixation Duration	0.45	0.58	0.29	0.59	0.56	0.54	s

Consistent with the previous observations, the AOI analysis also revealed a significant improvement in participants’ visual attention and cognitive processing abilities in the post-design scenes. At the design element level, the study found that, prior to the design modifications, participants paid more attention to the ground, walls, and greenery. Furthermore, the modification of the wall and plant elements significantly affected the participants’ physiological responses, with the average pupil diameters of the left and right eyes increasing from 3.90 mm and 3.95 mm pre-design to 3.96 mm and 3.97 mm post-design, respectively. This suggests that controlling and adjusting the building facades, as well as strategically placing greenery, help enhance the overall functionality and aesthetic value of the scene, thus improving visual appeal. These findings align with the results from the preliminary experiment, which emphasized the significant role of wall and green environment elements in shaping visual attention and environmental impressions [17]. The gaze duration on the water element significantly increased post-design, rising from 0.11 seconds to 0.59 seconds. This notable increase can likely be attributed to the visual fluidity and natural beauty of the water, which heightened the environment’s appeal. However, the average saccade velocity in the water region rose from 0.29 px/ms to 1.79 px/ms, indicating that participants were more actively exploring the visual scene, with more visual points of interest emerging after the modification.

#### The impact of spatial design elements on physiological responses.

The primary focus of eye movement analysis is to record and quantify an individual’s eye movement patterns while observing visual scenes, reflecting the subject’s allocation of attention and information processing strategies in rural spaces. However, eye movement data cannot directly reveal the subject’s cognitive state or physiological responses. Therefore, to explore the physiological mechanisms behind visual attention patterns, we used spatial scenes (pre-design and post-design) as the independent variable and HRV indices (SDNN, RMSSD) as the dependent variables, with paired sample t-tests for analysis. The results showed significant differences in HRV levels before and after spatial design, with SDNN (t = 6.640, p = 0.000) and RMSSD (t = -2.716, p = 0.011) indicating that the design transformation had an impact on heart rate variability. HRV, as a key indicator for assessing the functional activity level of the autonomic nervous system, provides a method for quantitative analysis. Based on the ECG data formula in section 2.5.2, heart rate data were converted into heart rate variability indices, enabling a detailed analysis of the impact of rural space renovation on the physiological states of residents before and after the transformation.

**Fig 8:**
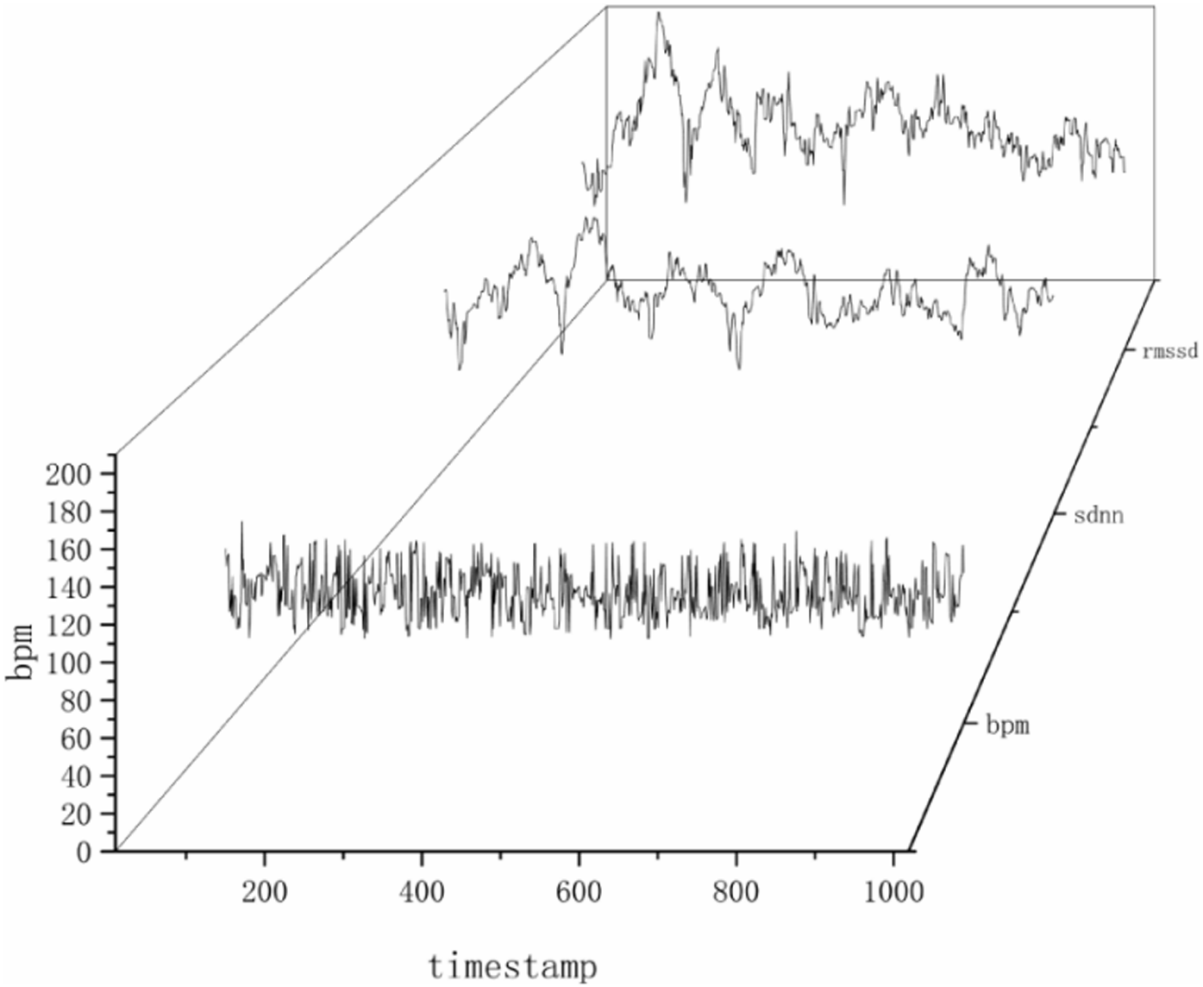
Conversion of heart rate data to heart rate variability indices.

Heart rate data are converted into heart rate variability indices (Fig 8). By transforming the bpm into SDNN and RMSSD curves, it is possible to observe how the pre- and post-renovation scenes affect the participants’ heart rate variability indices differently. The rural space renovation may influence residents’ physiological and psychological health by altering their living environment and daily activity patterns.

**Fig 9:**
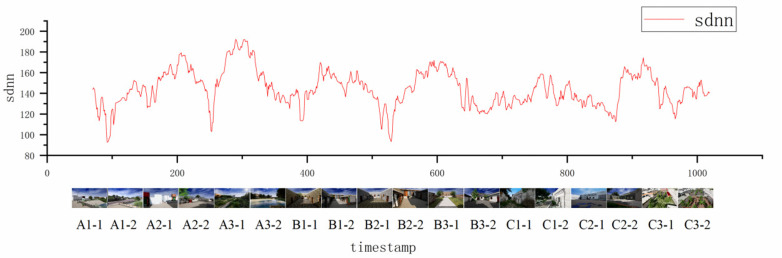
Comparison of SDNN and renovation scenes.

To assess how different rural space renovation scenes affect the participants’ heart rate variability index the SDNN curves are matched with the rural space scenes (Fig 9). After the renovation, scenes A1, B1, B2, B3, C1, C2, and C3 led to an increase in SDNN data, indicating that these scenes may enhance the residents’ autonomic nervous system regulation, particularly parasympathetic nervous activity. This is often associated with individuals being in a relaxed state and having improved adaptability to stress.

#### The impact of spatial design elements on subjective emotions.

By analyzing eye-tracking and ECG data, this study discusses the physiological responses of participants in different rural space renovation scenes, providing a quantitative perspective for assessing the impact of rural space renovation on residents’ emotional states. To gain a more comprehensive understanding of how rural space renovation affects residents’ comfort and emotions, spatial scenes (pre-design and post-design) were used as independent variables, with subjective questionnaire data as the dependent variables, and a Wilcoxon signed-rank test was conducted. The results indicate that the subjective questionnaire responses exhibited a statistically significant difference between pre- and post-design conditions (p ≈ 0.000 < 0.05), demonstrating a significant change in participants’ subjective emotional states before and after the design modifications.

**Fig 10:**
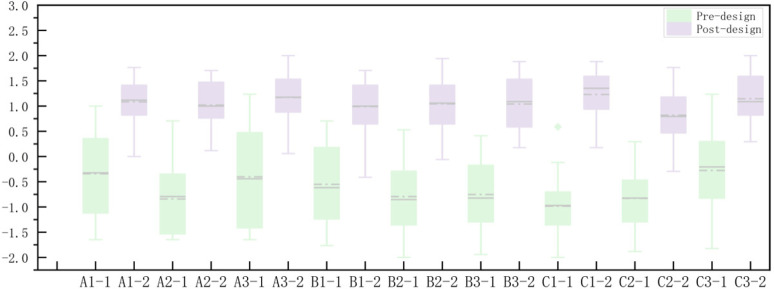
Box plot of subjective preference scores.

Using Origin2022 software, a box plot and line graph of subjective preference scores were created to discuss the changes in subjective evaluations of rural scenes before and after renovation. The graph presents the average subjective preference scores and their relationship with the pre- and post-renovation scenes (Fig 10). The results indicate that the subjective evaluations of all nine groups of rural scenes were higher after the renovation design, reflecting participants’ higher satisfaction and approval of the post-renovation environment.

#### Multidimensional correlation analysis of visual, physiological, and emotional data.

**Fig 11:**
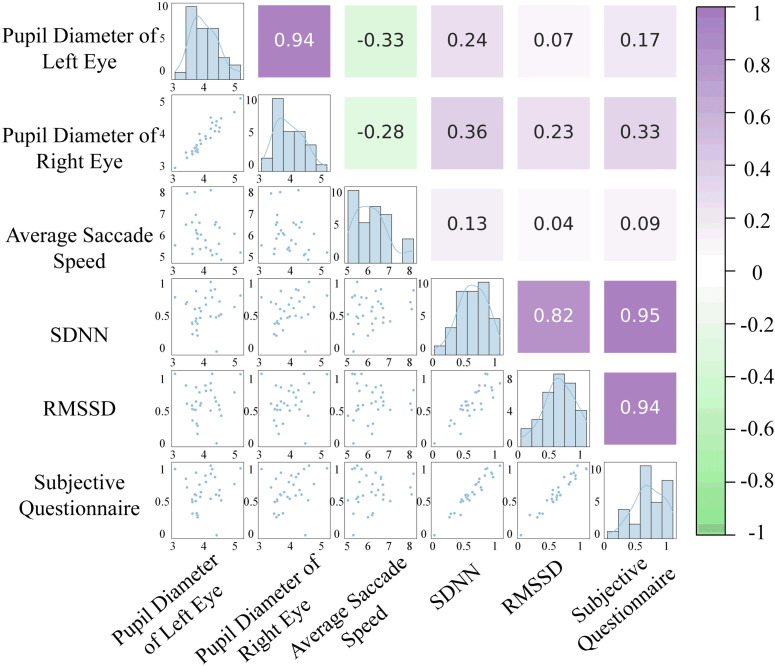
Correlation analysis.

To gain a deeper understanding of how rural space design affects emotional experiences before and after the renovation, the correlation between eye-tracking data, ECG data, and subjective preference was analyzed, discussing whether there is consistency between visual, physiological, and emotional data. As discussed in Section 2.6, the pupil diameter of the left and right eyes, average saccade velocity, SDNN, RMSSD, and subjective questionnaire data do not fully meet the normal distribution. Therefore, Spearman correlation tests were performed using Python 3.12.3 and PyCharm 2024.1.2 software to calculate the correlation coefficients. The correlation coefficient ρ_s_ ranges from -1 to + 1, with values close to ± 1 indicating a very strong correlation, 0.6 to 0.8 (or -0.6 to -0.8) indicating a strong correlation, 0.4 to 0.6 (or -0.4 to -0.6) indicating a moderate correlation, 0.2 to 0.4 (or -0.2 to -0.4) indicating a weak correlation, and values close to 0 indicating a very weak or no correlation [18]. The results of the correlation analysis are shown in Fig 11.

The results of the correlation analysis reveal important connections between physiological indicators, providing a scientific basis for a deeper understanding of individuals’ internal response mechanisms to environmental stimuli. The significant positive correlation (ρ_s_ = 0.94) between the left and right pupil diameters indicates that individuals’ eye responses are highly synchronized when exposed to visual stimuli. The strong correlation (ρ_s_ = 0.82) between the heart rate variability indicators SDNN and RMSSD reveals the complexity of heart rate fluctuations and their close relationship with vagal nerve regulation of the heart. Under the regulation of the autonomic nervous system, heart rate variability is closely linked with parasympathetic nervous activity, which may be associated with individuals’ adaptive responses to stress or relaxation. Additionally, the correlation coefficients between heart rate variability indicators and subjective questionnaire results were found to be ρ_s_ = 0.94 and ρ_s_ = 0.95, showing a very strong positive correlation. This finding provides new insight into the relationship between participants’ physiological responses and psychological states. As key indicators of autonomic nervous system activity, changes in heart rate variability can effectively reflect individuals’ abilities in emotional regulation. Therefore, a detailed discussion of visual eye-tracking indicators, heart rate variability indicators, and subjective questionnaire results can help explore the specific impacts of rural environmental stimuli on participants’ physiological and psychological states.

## Discussion

This study explores the comprehensive impact of rural space design on residents’ physiological, psychological, and emotional responses through the multidimensional analysis of eye-tracking data, HRV, and subjective emotional data. The results indicate that rural space renovation significantly improved residents’ visual attention, cognitive processing abilities, and emotional experiences.

### Analysis of the factors influencing visual attention

The study found that in terms of visual attention, the renovated rural spaces significantly enhanced participants’ pupil response, with most scenes showing notable improvements in visual appeal, cognitive complexity, attention guidance, and emotional stimulation. This phenomenon suggests that optimizing the aesthetic features of the environment not only effectively enhances the intensity of emotional experiences but may also increase cognitive load, requiring participants to devote more attention when processing visual information [34]. Changes in pupil diameter are often directly related to emotional responses, with pupil dilation typically being a physiological manifestation of the intensity of visual stimuli or emotional arousal [35]. A decrease in saccade speed is also closely linked to an increase in cognitive load, indicating that the brain requires more time to integrate information when faced with complex visual input [36]. This suggests that while the renovated rural environment improves visual appeal, it also increases the demand for cognitive resources.

The optimization of architectural facade design and the strategic placement of greenery not only enhance the functionality and aesthetic value of the space but also effectively guide participants’ visual focus. The addition of water elements significantly increased participants’ gaze duration, indicating that the visual fluidity and natural beauty of water enhanced the environment’s appeal. As part of the natural landscape, water is often regarded as possessing strong visual attraction due to its fluidity and variability, which can evoke deep emotional responses [37]. This visual fluidity not only stimulates the senses but also enhances the overall aesthetic of the environment, thereby strengthening the emotional effects and depth of memory associated with the space. When rural spaces introduce new aesthetic elements, these elements, as initial visual stimuli, can induce pupil dilation, prompting participants to gather more information [38]. Subsequently, as the brain processes this complex visual information, the increased complexity of information processing further heightens cognitive load. As research indicates, an increase in cognitive load often results in sustained pupil dilation, which is a typical physiological response during high-intensity information processing [39]. At the same time, the guiding role of design elements activates brain regions related to emotion, triggering emotional fluctuations and further intensifying the interaction between visual stimuli and emotions. Therefore, spatial design should not only focus on the combination of aesthetics and functionality but also strategically incorporate various design elements to guide attention, stimulate positive emotions, and avoid excessive cognitive load.

In summary, the design of rural spaces should find an accurate balance between aesthetics and functionality. While enhancing visual appeal and guiding attention, excessive cognitive complexity may have negative effects, leading to visual fatigue or emotional over-stimulation. Therefore, the renovation of rural spaces should carefully consider the appropriate complexity of visual elements in the design, aiming to meet aesthetic needs without unnecessarily increasing cognitive load due to overly complex design. In practice, designers should focus on the unity of the spatial interface, avoiding the excessive accumulation of different styles and elements, and ensuring coordination between architecture and natural landscapes. A unified interface helps form a clear visual path, reducing cognitive distractions, making the space more approachable and livable. Additionally, the thoughtful arrangement of vegetation should be based on the actual needs of the space and the characteristics of the environment. Selecting plants that are suitable for the local climate and cultural context enhances the natural feel and visual comfort of the space. Through rich layers and color variations, these elements can stimulate emotional responses and guide the flow of visual focus.

### The relationship between physiological responses and design

The significant impact of rural space renovation on heart rate variability suggests that environmental design not only improves visual and emotional experiences but also profoundly affects residents’ physiological and psychological health by altering their living environments and daily activity patterns. Studies have shown that environmental optimization can regulate the autonomic nervous system, particularly enhancing the activity of the parasympathetic nervous system, thereby promoting physical relaxation and balance [40]. In renovated rural settings, the optimization of environmental features through uniform interface styles, well-placed vegetation, and the subtle integration of cultural symbols stimulates sensory responses such as visual and auditory reactions. These sensory stimuli effectively activate the parasympathetic nervous system, leading to a reduction in heart rate and an increase in heart rate variability, ultimately helping the body enter a more relaxed and balanced state. This finding highlights the potential impact of rural space design on physical health. Rural space renovation should not be limited to aesthetic and functional considerations but should also prioritize physiological health needs as a core element of the design. During the rural design process, designers should focus on enhancing the naturalness and comfort of the environment while ensuring that the functionality of the space meets the daily life and recreational needs of residents. The configuration of plants, the increase in green spaces, and the proper setting of leisure areas not only enhance the aesthetic appeal of the space but also contribute to effective physiological and psychological regulation [41]. Furthermore, the integration of cultural symbols is a key factor in the design. By introducing design elements closely related to local culture and history, designers can not only strengthen residents’ sense of identity and belonging but also, through the creation of a cultural atmosphere, further stimulate positive emotional responses. This, in turn, promotes the activity of the parasympathetic nervous system, enhancing the overall health effects of the space.

Therefore, the renovation of rural spaces should fully consider the needs of physiological health. Specifically, during the design process, it is essential to consciously integrate environmental elements that promote physical and mental relaxation and physiological regulation. The design should focus on enhancing the naturalness, comfort, and functionality of the environment, implementing a health-oriented design philosophy. By increasing plant configurations, providing multifunctional recreational spaces, and strengthening the construction of cultural sites, the aesthetic experience of the space can be improved, while also promoting the physical and mental well-being of residents and the regulation of their physiological functions.

### Analysis of the impact of subjective emotions

The changes in rural space design have a profound impact on the physiological and psychological states of residents through multiple sensory channels. Research indicates that the renovated rural environment shows significant improvement at the subjective emotional level, with participants reporting a marked increase in satisfaction and sense of belonging after the transformation. This change suggests that when rural space design meets residents’ aesthetic needs, incorporates local characteristics and cultural connotations, and balances functionality with social needs, it tends to evoke positive emotional responses such as pleasure, satisfaction, and pride [42]. These emotional reactions are not only reflected in the aesthetic enjoyment of the environment but also in the reinforcement of residents’ sense of belonging to the space, further fostering positive psychological and emotional outcomes. Therefore, rural space design should fully consider the emotional needs of residents and create a unified design style through the thoughtful combination of various design elements, to establish an emotionally rich environment. Studies have shown that emotional design has a direct impact on human behavior and space interaction. Positive emotional experiences significantly increase the amount of time residents spend in a space, the frequency of their activities, and their level of interaction, thereby strengthening community cohesion [43]. These emotion-driven behavioral responses are crucial for the long-term development of a community. They not only help promote closer neighborly relationships but also create favorable conditions for the accumulation of social capital. Designers can further optimize residents’ emotional connections by incorporating cultural symbols and creating spaces that evoke emotional experiences, enhancing interaction and integration among community members. By emphasizing the uniqueness of local culture and the social interaction functions within the design, rural spaces can not only provide functional areas for residents but also create environments that resonate emotionally with them. This approach effectively promotes the social and cultural integration of the community [44].

### Comprehensive analysis of data correlation

Through a multidimensional correlation analysis of visual, physiological, and emotional data, this study reveals the high consistency between these data, providing a scientific basis for further understanding the intrinsic response mechanisms of individuals to environmental stimuli. Specifically, a significant positive correlation was found between the pupil diameter of the left and right eyes, indicating a clear synchrony between the two eyes during visual responses, which is closely related to both the visual input from both eyes and the brain’s processing mechanisms [45]. Additionally, the strong correlation between heart rate variability indicators, SDNN and RMSSD, reflects the close connection between heart vagus nerve regulation and heart rate variability, while the complexity of heart rate changes reveals the autonomic nervous system’s response mechanism to environmental stimuli [46]. Furthermore, the high positive correlation between heart rate variability and subjective questionnaire results further emphasizes the close relationship between physiological responses and psychological states, supporting the dual role of environmental factors in emotional and physiological responses [47]. The comprehensive analysis suggests that the impact of rural space design on residents is multidimensional. Visual, physiological, and emotional responses are not only interrelated but also influence each other. Within the framework of environmental psychology and neuroscience, the design elements of rural spaces, as environmental stimuli, activate the individual’s visual system, and through subsequent brain processing, trigger neural networks related to cognition, emotions, and physiological responses, thereby influencing the overall experience of residents.

In summary, this study reveals the multidimensional impact of rural space design elements on residents by comprehensively analyzing the relationships between visual, physiological, and emotional data. Future rural design should focus on how biological data can guide design decisions. Designers can optimize visual design elements to unify building facades, appropriately arrange vegetation and water features, thus enhancing visual appeal while avoiding overly complex designs. This can reduce cognitive load and enhance the positivity of emotional experiences. In terms of physiological regulation, the design should emphasize the integration of natural elements and comfortable environments to promote the activity of the parasympathetic nervous system, helping residents relax and improve their health. Furthermore, rural design should address the emotional needs of residents by incorporating cultural symbols and creating emotional spaces to strengthen community cohesion, enhance emotional experiences, and encourage social interaction. By combining scientific data analysis with design optimization, rural spaces can create a healthier, more livable, and vibrant community atmosphere, providing residents with a higher quality living environment and offering actionable evaluation methods for design. However, as this study did not include local villagers as experimental participants, it limits a deeper understanding of the emotional impact of rural space renovations on indigenous residents. Future research should pay more attention to the subjectivity of rural residents, fully considering their needs and feedback, and adopt a multi-stakeholder participatory approach to obtain a more comprehensive and in-depth evaluation perspective.

## Conclusion

Rural space design is not only the shaping of physical form, but also the inheritance of social relationship and cultural meaning. In the face of problems such as weak infrastructure construction and homogenization of rural environment, it is necessary to pay attention to functionality and aesthetics in design, and more importantly, it is necessary to dig deeper into the cultural characteristics and social needs of the countryside, so as to promote the reorganization of resources, the reconstruction of relations and the reshaping of values in the governance of rural space through the spatial reshaping and the spatial production mechanism of cultural potentials. In this process, the understanding of rural spatial emotions is crucial.

This study, through experimental design and data analysis, quantifies emotional states and explores the impact of rural space renovations on emotional changes. The results indicate that modifications in rural space design can significantly improve residents’ visual attention, emotional experiences, and physiological relaxation. By strategically arranging vegetation, optimizing spatial interfaces, and incorporating cultural symbols, the renovated rural spaces showed notable improvements in visual appeal, emotional experiences, psychological relaxation, and overall resident satisfaction. Through adjustments in the layout of vegetation, treatment of spatial interfaces, and the integration of cultural symbols, the redesigned rural environment demonstrated significant improvements in cognitive attention, emotional positivity, and psychological relaxation, as reflected in visual data, ECG data, and subjective evaluations from the participants. The post-renovation rural spaces effectively enhanced visual attractiveness, emotional experiences, psychological relaxation, and increased resident satisfaction. Therefore, the spatial renovation measures adopted were effective and had a positive impact on the space, leading to substantial improvements in residents’ overall experiences.

In summary, by comparing the results of the data, the researcher can more accurately understand the needs of the subjects and identify which design elements can elicit positive emotional responses. The combination of eye-tracking data, ECG data and subjective evaluation questionnaires can help designers better understand the user experience and improve the effectiveness and acceptance of design, providing a scientific basis for rural environment design. Future research should further explore how design elements affect human emotional and behavioral responses, and achieve sustainable development of rural spaces and maximize the well-being of residents through an interdisciplinary approach.

## Supporting information

S1 File Supplementary raw data file. (RAR)

## References

[1] Wang YC, Liu BY. On Chinese rural landscape and rural landscape planning. China Landscape Archit. 2003; 01:56-59.

[2] Sun X. Landscape architecture aesthetics. China Landscape Architecture. 1992; 8(2):9.

[3] Grinde B, Patil GG. Biophilia: does visual contact with nature impact on health and well-being?. Int J Environ Res Public Health. 2009;6(9):2332–2343. doi: 10.3390/ijerph6092332 19826546 PMC2760412

[4] Hsueh B, Chen R, Jo Y, Tang D, Raffiee M, Kim YS, et al. Cardiogenic control of affective behavioural state. Nature. 2023;615(7951):292–299. doi: 10.1038/s41586-023-05748-8 36859543 PMC9995271

[5] Cao J, Zhang M. Evaluation of urban waterfront landscape quality based on semantic differential method: a case of Zhonghuamen section of Qinhuai River in Nanjing. J Nanjing For Univ. 2020; 44(6):221.

[6] Emotional engineering. Springer; 2016.

[7] Ren H, Shi M, Zhang J. Research contents, methods and prospects of emotional architecture based on a systematic literature review. Buildings. 2024;14(4):997. doi: 10.3390/buildings14040997

[8] Chao G, Ying Z, Kang W. Research advances and prospects of eye tracking. Acta Autom Sinica. 2022; 48(5):1173-1192.

[9] Fu H, Xue P. Cognitive restoration in following exposure to green infrastructure: an eye-tracking study. J Green Build. 2023;18(2):65–88. doi: 10.3992/jgb.18.2.65

[10] Fu H, Tan Y, Xia Z, Feng K, Guo X. Effects of construction workers’ safety knowledge on hazard-identification performance via eye-movement modeling examples training. Saf Sci. 2024; 180:106653.

[11] Su T, Wang K, Li S, Wang X, Li H, Ding H, et al. Analysis and optimization of landscape preference characteristics of rural public space based on eye-tracking technology: the case of Huangshandian village, China. Sustainability. 2022;15(1):212. doi: 10.3390/su15010212

[12] Zhang F, Haddad S, Nakisa B. The effects of higher temperature setpoints during summer on office workers’ cognitive load and thermal comfort. Build Environ. 2017; 123:176-188.

[13] Yeom S, Kim H, Hong T. Psychological and physiological effects of a green wall on occupants: a cross-over study in virtual reality. Build Environ. 2021;204 108134

[14] Wang X, Liu S, Zhu M. Flexible non-contact electrodes for wearable biosensors system on electrocardiogram monitoring in motion. Front Neurosci. 2022;16:900146. doi: 10.3389/fnins.2022.900146 35747208 PMC9209699

[15] Wang Y, Li J, Wang H. Non-contact wearable synchronous measurement method of electrocardiogram and seismocardiogram signals. Rev Sci Instrum. 2023; 94(3). 10.1063/5.0120722 37012744

[16] Bedon C, Mattei S. Remote facial expression and heart rate measurements to assess human reactions in glass structures. Adv Civil Eng. 2021; 2021(1):1978111.

[17] Ren H, Zhang L, Zhang J, Wang X, Wang Q. Exploration of a rural street environment: the difference in sight between villagers and tourists. Sustainability. 2024;16(7):2653. doi: 10.3390/su16072653

[18] Cohen J. Statistical power analysis for the behavioral sciences. Routledge; 2013.

[19] Shach-Pinsly D, Shadar H. The public open space quality in a rural village and an urban neighborhood: a re-examination after decades. Sustainability. 2024;16(18):7938. doi: 10.3390/su16187938

[20] Luan F, Pei Z, Cao S. Practical rural landscape planning: methods and practical exploration. Urban Plann Forum. 2022; (03):65-71. doi: 10.16361/j.upf.202203009

[21] Yamashita R, Chen C, Matsubara T, Hagiwara K, Inamura M, Aga K, et al. The mood-improving effect of viewing images of nature and its neural substrate. Int J Environ Res Public Health. 2021;18(10):5500. doi: 10.3390/ijerph18105500 34065588 PMC8161053

[22] Gorin H, Patel J, Qiu Q, Merians A, Adamovich S, Fluet G. A review of the use of gaze and pupil metrics to assess mental workload in gamified and simulated sensorimotor tasks. Sensors. 2024;24(6):1759. doi: 10.3390/s24061759 38544022 PMC10975796

[23] Takahashi M, Kikuchi M, Yamamoto J. Gaze and avoidant patterns of visual attention to aversive stimuli during fear habituation trial: a pilot eye tracking study. J Behav Cogn Ther. 2023; 33(4):227-235.

[24] Nicolas J, Bidet-Caulet A, Pélisson D. Reactive saccade adaptation boosts orienting of visuospatial attention. Sci Rep. 2020; 10(1):13430. 32778710 10.1038/s41598-020-70120-z PMC7417993

[25] Kang OE, Huffer KE, Wheatley TP. Pupil dilation dynamics track attention to high-level information. PLoS One. 2014;9(8):e102463. doi: 10.1371/journal.pone.0102463 25162597 PMC4146469

[26] Zeid S, Buch G, Velmeden D, Söhne J, Schulz A, Schuch A, et al. Heart rate variability: reference values and role for clinical profile and mortality in individuals with heart failure. Clin Res Cardiol. 2024;113(9):1317–30. doi: 10.1007/s00392-023-02248-7 37422841 PMC11371886

[27] Shaffer F, Ginsberg JP. An overview of heart rate variability metrics and norms. Front Public Health. 2017;5:258. doi: 10.3389/fpubh.2017.00258 29034226 PMC5624990

[28] Zhuang WM. SD method and architectural space environment evaluation. J Tsinghua Univ (Sci & Tech).1996;(04):42-47. doi: 10.16511/j.cnki.qhdxxb.1996.04.009

[29] Likert R. A technique for the measurement of attitudes. Arch Psychol. 1932; 140:1–55

[30] Hanusz Z, Tarasińska J. Normalization of the Kolmogorov–Smirnov and Shapiro–Wilk tests of normality. Biometrical Lett. 2015;52(2):85–93. doi: 10.1515/bile-2015-0008

[31] Kim TK. T test as a parametric statistic. Korean J Anesthesiol. 2015;68(6):540–546. doi: 10.4097/kjae.2015.68.6.540 26634076 PMC4667138

[32] MacFarland TW, Yates JM, MacFarland TW. Wilcoxon matched-pairs signed-ranks test. In: Introduction to nonparametric statistics for the biological sciences using R. 2016; 133-175.

[33] Cattaneo T, Giorgi E, Ni M. Landscape, architecture, and environmental regeneration: a research by design approach for inclusive tourism in a rural village in China. Sustainability. 2018;11(1):128. doi: 10.3390/su11010128

[34] Zhou X, Cen Q, Qiu H. Effects of urban waterfront park landscape elements on visual behavior and public preference: evidence from eye-tracking experiments. Urban Forestry & Urban Greening. 2023;82:127889. doi: 10.1016/j.ufug.2023.127889

[35] Snowden RJ, O’Farrell KR, Burley D, Erichsen JT, Newton NV, Gray NS. The pupil’s response to affective pictures: role of image duration, habituation, and viewing mode. Psychophysiology. 2016;53(8):1217–1223. doi: 10.1111/psyp.12668 27172997 PMC5031225

[36] Oyama A, Takeda S, Ito Y, Nakajima T, Takami Y, Takeya Y, et al. Novel method for rapid assessment of cognitive impairment using high-performance eye-tracking technology. Sci Rep. 2019;9(1):12932. doi: 10.1038/s41598-019-49275-x 31506486 PMC6736938

[37] Mahmood T, Wall RS, Ballinas MBP. Happy blue: the impact of water-bodies on happiness by visual perception of human defining landscape aesthetic in context of Rotterdam. 2015.

[38] Ren H, Yang F, Zhang J. Evaluation of cognition of rural public space based on eye tracking analysis. Buildings. 2024;14(6):1525. doi: 10.3390/buildings14061525

[39] Weber P, Rupprecht F, Wiesen S. Assessing cognitive load via pupillometry. In: Advances in artificial intelligence and applied cognitive computing: proceedings from ICAI’20 and ACC’20. Springer International Publishing; 2021. p. 1087-1096.

[40] Mobbs D, Hagan CC, Dalgleish T, Silston B, Prevost C. The ecology of human fear: survival optimization and the nervous system. Front Neurosci. 2015;9:121062. doi: 10.3389/fnins.2015.00055 25852451 PMC4364301

[41] Ma X, Yan T, Qin Y, Wang H, Yan Y. Optimization of green space plant configuration in residential areas of Chongqing central business district based on green plot ratio—a case study of Xuhui city residential community. J Geosci Environ Protect. 2023; 11(6):37-49.

[42] Chen G, Yan J, Wang C, Chen S. Expanding the associations between landscape characteristics and aesthetic sensory perception for traditional village public space. Forests. 2024;15(1):97. doi: 10.3390/f15010097

[43] Wang S, Inkuer A, Mayusoh C, Puntien P. The power of emotional design: a study on visual interface design to enhance user engagement in online exhibition platforms. Int J Multidiscip Stud. 2024; 1201.

[44] Mamajonova N, Oydin M, Usmonali T. The basics of architectural design. Holders of Reason. 2024; 2(1):369-382.

[45] Honnuraiah S, Huang HHY, Ryan WJ. Cellular and circuit mechanisms underlying binocular vision. bioRxiv. 2024: 2024.03.11.584536.

[46] Salazar-Martínez E, Naranjo-Orellana J, Sarabia-Cachadiña E. Heart rate variability: obtaining the stress score from SDNN values. Isokinet Exerc Sci. 2024; 32(4):301-307.

[47] Beatton T, Chan HF, Dulleck U. Positive affect and heart rate variability: a dynamic analysis. Sci Rep. 2024;14(1):7004. doi: 10.1038/s41598-024-57279-5 38523154 PMC10961327

